# Analysis of salivary proteomic biomarkers for the surveillance of changes in high-risk status of early childhood caries

**DOI:** 10.1186/s12903-021-01930-4

**Published:** 2021-11-08

**Authors:** Xinzhu Zhou, Haozhe Li, Ce Zhu, Chao Yuan, Chunhua Meng, Shulan Feng, Xiangyu Sun, Shuguo Zheng

**Affiliations:** 1 Department of Preventive Dentistry, Peking University School and Hospital of Stomatology, National Center of Stomatology, National Clinical Research Center for Oral Diseases, National Engineering Laboratory for Digital and Material Technology of Stomatology, Beijing, 100081 PR China; 2 Department of Preventive Dentistry, Shanghai Jiao Tong University School of Dentistry, Shanghai Ninth People’s Hospital, Shanghai, 200011 PR China; 3 Second Kindergarten in Asian Games Village, Chaoyang Disctrict, Beijing, Beijing, 100101 PR China

**Keywords:** Early childhood caries, High risk, Saliva, Proteomic biomarker

## Abstract

**Background:**

Early childhood caries is an urgent public health concern. The aim of this study was to investigate salivary proteomic biomarkers for the surveillance of changes in the high-risk status of early childhood caries. The process involves the screening of specific salivary peptides that were differentially expressed only under dynamic changes in individual caries status.

**Methods:**

Stimulated whole saliva samples were collected from 28 kindergarten children aged 3–4 years in Beijing at baseline and 3 months and 6 months after baseline. A total of 68 samples were collected. In terms of their caries status and progress during the observation period, participants were divided into 3 groups; 7 in the non-caries recurrence group, 6 in the caries recurrence group, and 15 in the healthy control group. Salivary peptides that exhibited no significant differences in cross-sectional comparisons between different groups of caries status but only expressed differentially along with dynamic changes of individual caries were screened using the technique of magnetic beads combined with matrix-assisted laser desorption-ionization time-of-flight mass spectrometry (MALDI-TOF MS). The technique of liquid chromatography-electrospray ionization-tandem mass spectrometry (LC-ESI-MS/MS) was employed to identify the proteins from which these peptides were derived.

**Results:**

We found two salivary peptides differentially expressed only under dynamic changes in individual caries status in the above comparisons; mass-to-charge ratio (*m*/*z*) values of the two peptides were 1045.9 and 2517.6, respectively (*P* < 0.05). Principal component analysis (PCA) and the decision tree model based on these two peptides showed an acceptable distinguishing ability for changes in the high-risk status of early childhood caries. The source proteins of the two peptides with *m*/*z* values of 1045.9 and 2517.6 were identified as submandibular gland androgen regulatory protein 3B (SMR-3B) and mucin-7, respectively.

**Conclusions:**

Two proteins in children’s saliva, namely SMR-3B and mucin-7, have the potentiality to serve as candidate biomarkers for dynamic surveillance of changes in high-risk status of early childhood caries.

## Background

Early childhood caries is one of the most common chronic diseases in children, affecting the oral health status of more than 600 million children worldwide and posing a significant challenge to the global public health field [1]. The fourth national oral epidemiological survey in China found that the prevalence of dental caries among 5-year-old children was 71.9%, which showed a rapid growth trend from previous surveys, and 75.4% of caries were concentrated in one-third of these children [2]. Dental caries in children have the characteristics of early onset, rapid progress, and great harm. It is one of the main risk factors for inheriting permanent dentition caries [3]. Many factors, including oral hygiene habits, dietary habits and family environment, influence caries in children [4]. Additionally, children may have a higher risk for future caries after treatment [5, 6]. Untreated caries has a significant impact on children's chewing, pronunciation, and growth. General anaesthesia can be used in specific settings when children have extensive tooth decay, which ultimately causes a considerable burden on the family and society [7, 8]. Prevention and early treatment of dental caries is an important guarantee for maintaining children's oral and general health. Early screening of high-risk groups and timely targeted measures to prevent or reduce the occurrence of dental caries should be considered with high priority.

Risk assessment of caries at an early stage is a critical process to reduce the endangerment associated with the progression of advanced caries. At present, caries assessment models based on salivary protein, microorganisms and biochemical factors [9–11], as well as the microstructure and gene level of tooth enamel [12, 13], have exhibited a good ability to evaluate the risk of childhood caries. By means of effectively screening caries in high-risk populations, the individualized caries risk assessment model can provide a theoretical basis for the formulation of oral public health and health education and facilitate the rational allocation of oral health care resources.

A variety of proteins and peptides in saliva play an important role in maintaining and regulating the homeostasis of the oral environment and could be used to monitor the dynamic changes in oral health and disease states. Studies have shown that salivary proteins can form a protective barrier on the surface of teeth to formulate a defence system to inhibit demineralization and attract deposition of calcium and phosphorus ions to promote enamel remineralization [14]. Meanwhile, salivary proteins have the ability to interact with microorganisms to inhibit cariogenic bacterial aggregation and maintain oral stability [15]. As a result of the advantages of being non-invasive, fast, easy to transport and store, saliva has become a crucial medium for the study of caries biomarkers. As the key to preventing caries in children, the application of salivary protein biomarkers for early screening of high-risk groups has realistic significance and research value.

Currently, research on saliva biomarkers mostly reports differentially expressed proteins or peptides by comparing the status of specific caries, whereas few studies have attempted to explore dynamic changes in individual caries status. Therefore, further application of these candidate biomarkers is still in doubt. This study investigates salivary proteins and peptides that show no significant differences in the cross-sectional comparisons between different groups but are only differentially expressed along with dynamic changes in individual caries status. These potential biomarkers will definitely contribute to surveillance of changes in high-risk status of early childhood caries more accurately and effectively. Consequently, more effective prevention and intervention measures could be implemented for these groups with a higher risk of caries, approaching the goal of comprehensive prevention and treatment of early childhood caries focusing on the key population in the future.

## Methods

### Recruitment of participants, oral examination and follow-up

In this study, 28 children aged 3–4 years from a kindergarten in Beijing were enrolled with informed consent provided by their parents. The protocol and other related materials were approved by the Institutional Review Board of Peking University School of Stomatology (Issuing number: PKUSSIRB-201735057). All procedures were in accordance with the Declaration of Helsinki.

Oral examination was completed by an experienced clinical dentist, and his own standard consistency test showed a Kappa value higher than 0.85. The caries diagnoses were in accordance with the relevant standards about caries diagnostic criteria of the World Health Organization (5^th^ edition, 2013). Diagnostic criteria for dental caries used in this study deemed that a lesion in a pit or fissure or on the smooth surface of a tooth that has undermined enamel, a prominent cavity, or a softened cavity could be regarded as a caries. The inclusion criteria were as follows: (1) no upper respiratory tract infectious disease or antibiotics taken within the past month; (2) no systemic disease; (3) no mucosal disease or maxillofacial surgery related to disease in the oral cavity; and (4) compatible sampling and treatment. Among all the children who underwent oral examination, only 28 met all the inclusion criteria and were finally enrolled in the present study.

All participants were given oral hygiene and diet instructions during the 6-month follow-up period, after which all of them were divided into one of the following three groups according to caries status and progress of caries in the last 6 months: non-caries-recurrence group (Group C), caries-recurrence group (group CR), and healthy control group (Group H).

### Sample collection of saliva

The sampling time points in this study were the same as those for oral examination and follow-up, including baseline (T0), 3 months (T1), and 6 months (T2). Oral examination and saliva collection were performed at each time point, and the designated code was used to record the child’s caries status at that time point (0, absence of caries or caries experience; 1, presence of untreated caries; 2, presence of caries with history of treatment; 3, absence of caries with history of treatment; 4, presence of teeth missing due to caries). Whenever new carious lesions were found, they were properly treated within a short time period. Stimulated whole saliva samples were collected from 9:00 to 11:00 in the morning. Children were requested not to consume food or water within 1 h and rinse their mouth with clean water 10 min before the sampling procedures. A total of 3 ml of stimulated saliva samples (by brushing the occlusal surfaces of teeth only) were collected into a 5 ml tube. Each of the tubes was marked with a serial number and then immediately placed on ice. These saliva samples were sent to the laboratory within 4 h. Saliva samples were centrifuged at 10,000 rpm for 10 min at 4 °C. One millilitre of supernatant was transferred into a 1.5 ml EP tube and stored in a refrigerator at − 80 °C for further use.

### Peptidomic detection of saliva samples using mass spectrometry techniques

Magnetic bead kits (Bioyong Tech, Inc., Beijing, China) were used to analyse saliva samples. Magnetic beads combined with matrix-assisted laser desorption/ionization time of flight mass spectrometry (MALDI-TOF MS) were used for further analysis. The concrete procedure referred to the research of Si [16] and Sun [17]. A three-peptide mixture (monoisotopic molecular weight of 1533.8582, 2465.1989 and 5730.6087 Da; Sigma product numbers P2613, A8346 and I6279, respectively) was employed to calibrate the mass spectrometer. Each sample was analysed in triplicate in the MALDI procedures, while the subsequent analyses were based on the average spectra for each sample obtained by three-time repeated measurements using a merge calculator program (Bioyong Tech, Beijing, China). Then, mixed saliva samples were analysed using liquid chromatography-electrospray ionization-tandem mass spectrometry (LC-ESI-MS/MS). Proteomic analysis software Proteome Discoverer 2.1 (Thermo Fisher Technology Co., Waltham, MA, USA) was used to identify the source of the target peptide.

### Statistical analyses

One-way analysis of variance, chi-square test, Wilcoxon rank correlation test, and Kruskal–Wallis nonparametric test were performed using BioExplorer 1.0 software (Bioyong Tech, Inc.) and SPSS 25.0 software (IBM, Chicago, USA) to screen these peptides, which exhibited no significant differences in cross-sectional comparisons of the caries status between groups but only expressed differentially along with dynamic changes of individual caries. R Studio 1.4 software (R Studio Software Company, Boston, MA, USA) coupled with the R language 3.6.3 version package was used to draw principal component analysis diagrams. In this study, *P* < 0.05 was considered statistically significant.

## Results

### Characteristics of participants

The demographics of the three groups in the study [caries-free group (C group), caries-recurring group (CR group), healthy control group (H group)] are shown in Table 1. At baseline, the age (*P* = 0.205) and sex (*P* = 0.358) of the individuals participating in the study were not significantly different among all groups.

**Table 1:** Participant information

Group	Number of participants	Age (months)	Gender (M/F)	Caries experience	Caries recurrence
C	7	54.7 ± 8.8	2/5	√	×
CR	6	52.0 ± 7.0	4/2	√	√
H	15	57.7 ± 5.4	5/10	×	×

### Two peptides differentially expressed only under dynamic changes in caries status

A total of 68 saliva samples were collected at each time point during the study course. Using MALDI-TOF MS technology to compare the differences in polypeptide expression between different caries status groups, we found that 37 peptides showed no significant differences in any of the cross-sectional comparisons between various caries status groups.

**Fig. 1:**
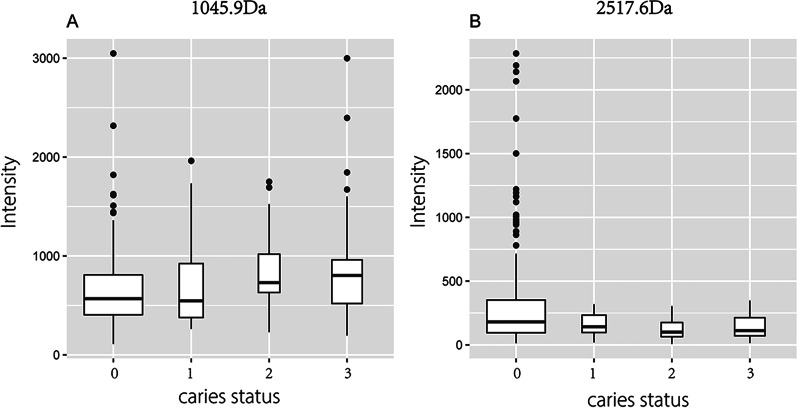
Comparison of peptides with m/z values of 1045.9 (**A**) and 2517.6 (**B**) among different caries statuses (*P* > 0.05); 0, absence of caries or caries experience; 1, presence of untreated caries; 2, presence of caries with history of treatment; 3, absence of caries with history of treatment; 4, presence of tooth missing due to caries

**Fig. 2:**
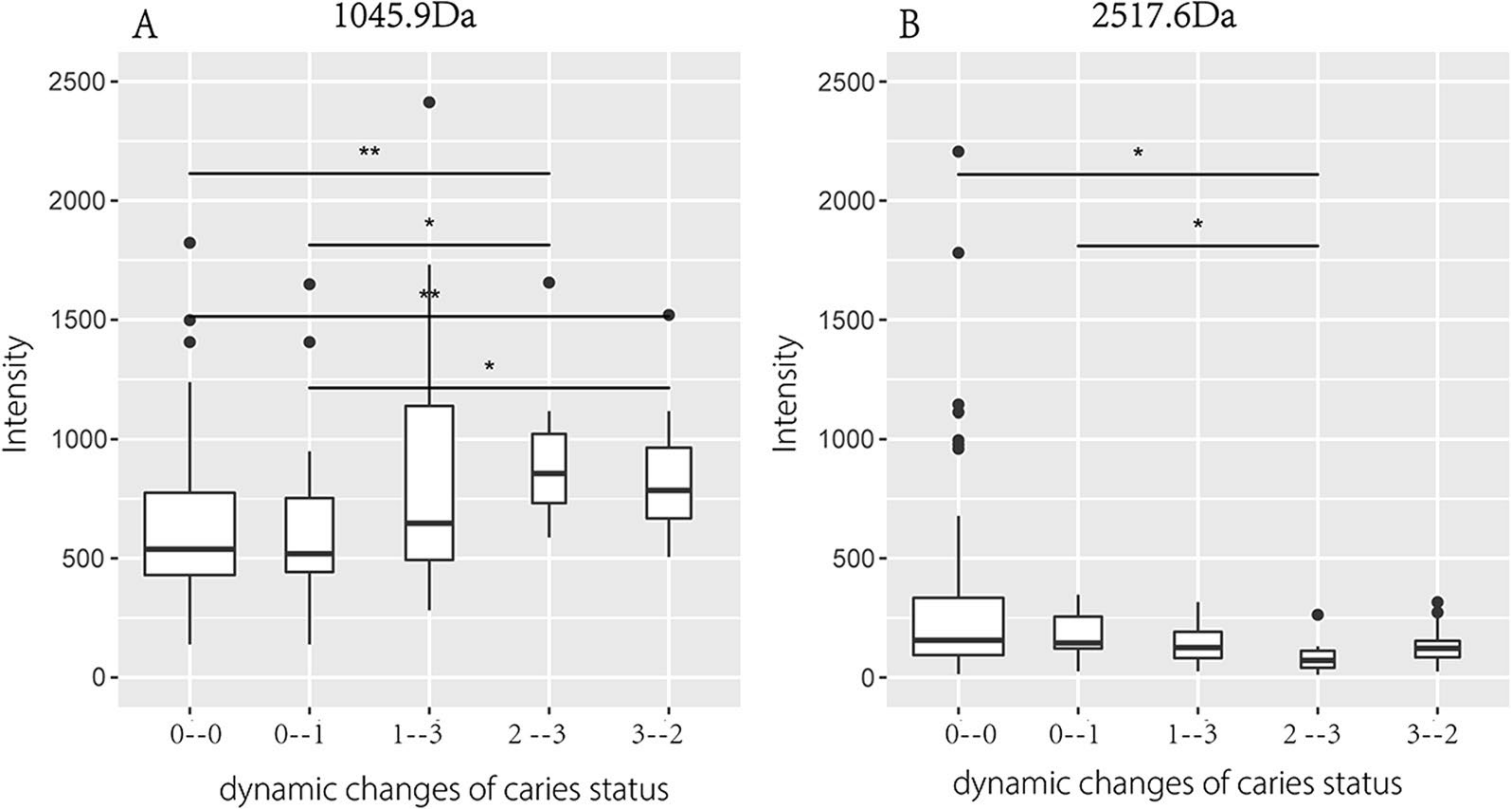
Comparison of peptides with *m/z* values of 1045.9 (**A**) and 2517.6 (**B**) under dynamic changes in caries status (*P < 0.05; **P < 0.01); 0–0, caries-free and have no caries experience; 0–1, Caries-free changes to caries and untreated; 1–3, caries and untreated changes to no caries after filling treatment; 2–3, caries after filling treatment, changes to no caries after filling treatment; 3–2, no caries after filling treatment changes to caries after filling treatment

**Fig. 3:**
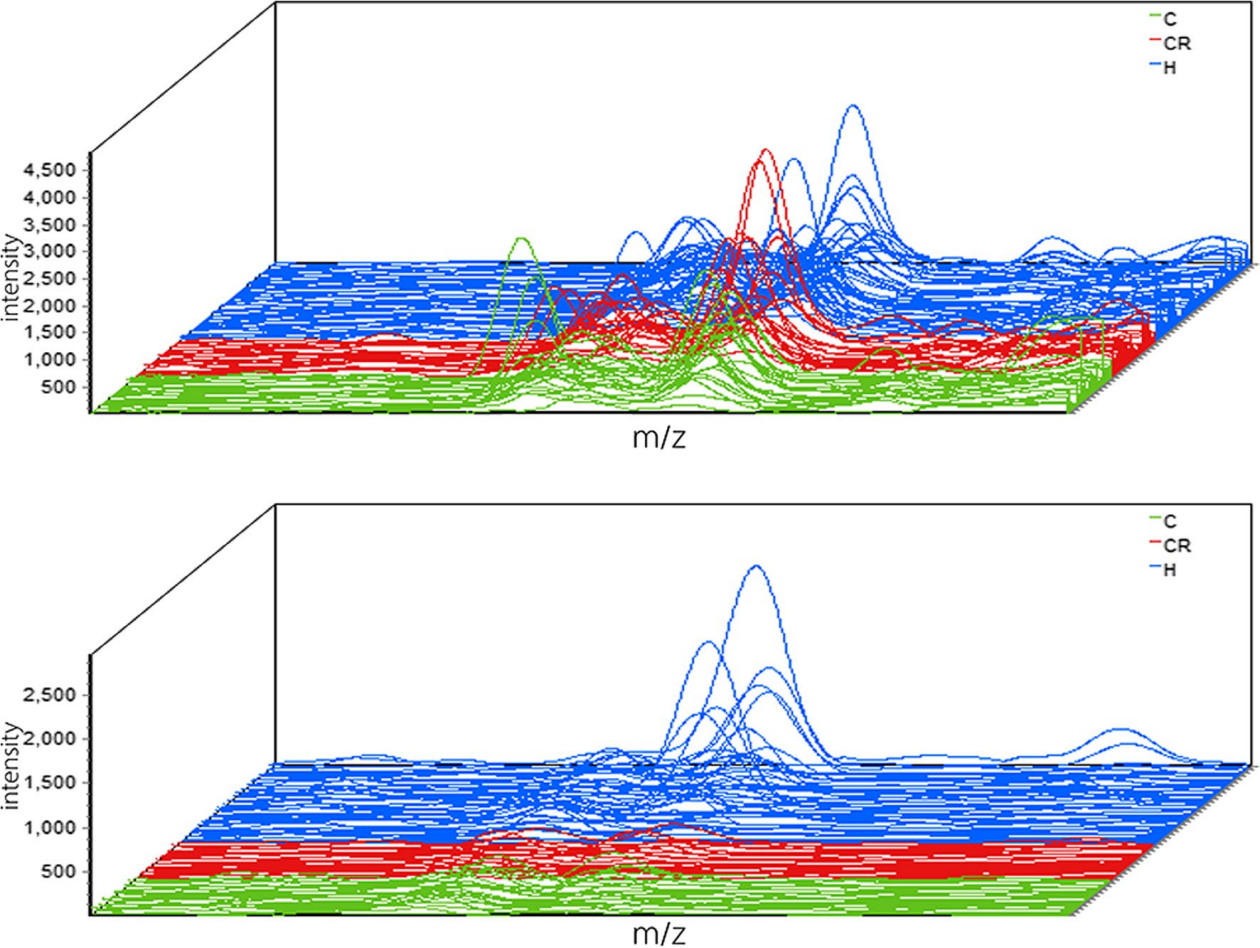
Three-dimensional intensity maps show two significantly different peptides with *m/z* values of 1045.9 and 2517.6

Further intergroup screening of the peptides was conducted based on the dynamic change characteristics of caries at each time point. As a result, two peptides, whose mass-to-charge ratio (*m/z*) values were 1045.9 and 2517.6, respectively (Figs. 1, 2, 3), were differentially expressed only with dynamic changes in individual caries status (*P* < 0.05) (Figs. 1, 2).

### Identification of the differentially expressed peptides

According to the LC–ESI–MS/MS results, the peptide with a m/*z* value of 1045.9 was identified as a segment of submaxillary gland androgen regulated protein 3B (SMR-3B), and the peptide with a m/*z* value of 2517.6 was identified to be derived from mucin-7 (Table 2).

**Table 2:** Identification of protein sources among differential peptides

*m/z* values	Identified proteins	Peptide sequence
1045.9	Submaxillary gland androgen regulated protein 3B (SMR-3B)	GIFPPPPPQP
2517.6	Mucin-7	SHFELPHYPGLLAHQKPFIRK

### Dispersion tendency of the three groups revealed by principal component analysis

**Fig. 4:**
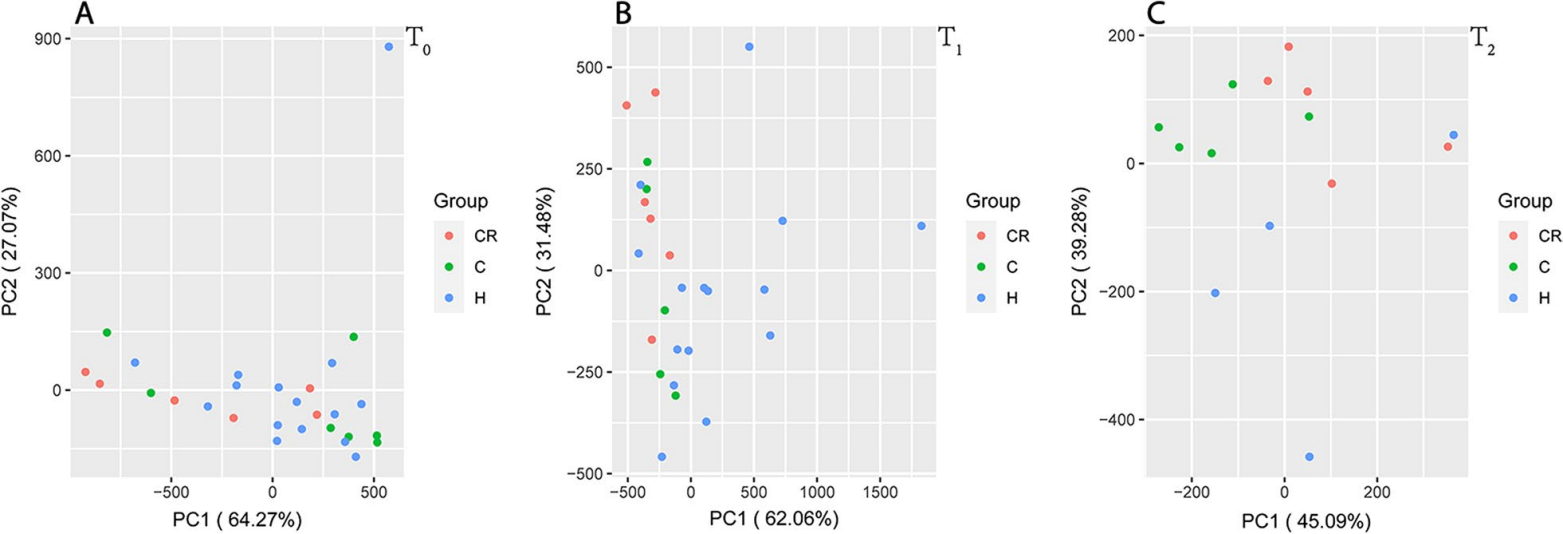
Principal component analysis of the three groups, namely, Group C (red), group CR (green) and Group H (blue) at the three time points of T0 (**A**), T1 (**B**) and T2 (**C**), respectively

Principal component analysis (PCA) was performed among the three groups at three time points based on these two peptides with *m*/*z* values of 1045.9 and 2517.6, respectively. The results showed that the dispersion tendency of the three groups was weaker at baseline (T0); the C + CR group and H group had a significant trend of separation at the time point of 3 months (T1). At 6 months (T2), there was a more dispersed state among the three groups (Fig. 4).

### Establishment of a decision tree to distinguish changes in high-risk caries status

**Fig. 5:**
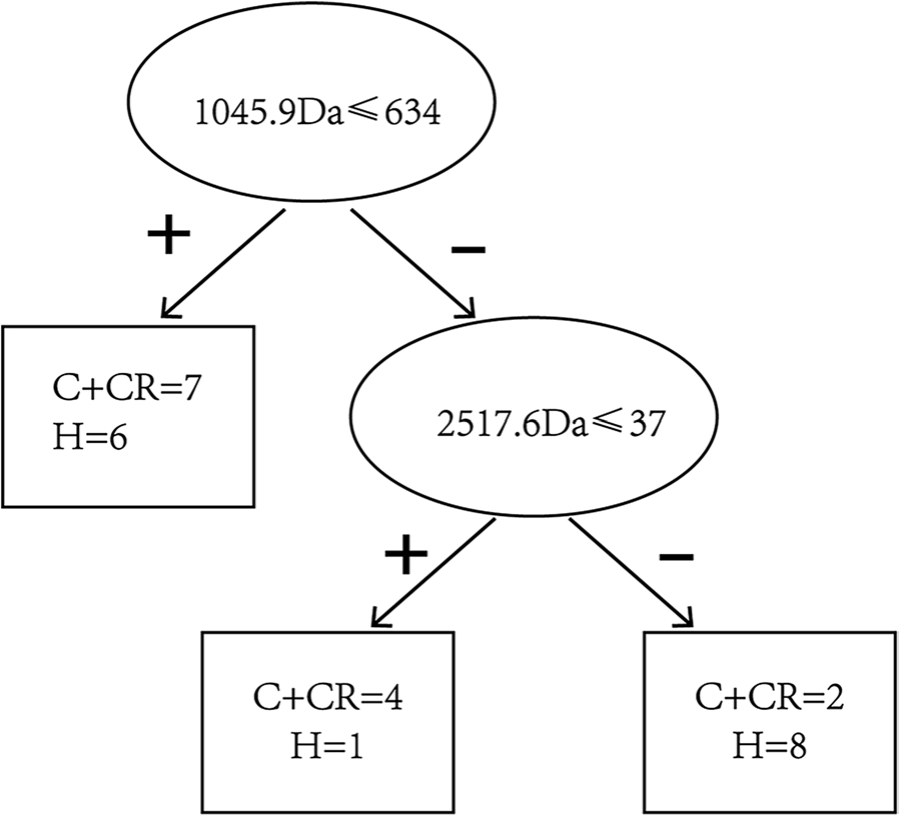
Decision tree analysis, in which decision nodules (oval) show differences based on the peak intensity of peptides, and status nodules (rectangular) represent the distribution of people between the high-risk status of caries (the combined group of C and CR) and caries-free status (Group H) after screening

According to the difference in peptide peak intensity, a decision tree model was established to screen individuals at high risk of caries (Fig. 5). When the peptide with the *m*/*z* value of 1045.9 showed an intensity of 634 or lower or the peptide with the *m*/*z* values of 1045.9 had an intensity of more than 634 but the intensity of the peptide with the *m*/*z* value of 2517.6 was 37 or lower, the individuals might fall into a group at a high risk of suffering from caries or caries recurrence, which could contribute to changes in the high-risk status of early childhood caries. Meanwhile, individuals with a peptide that had an m/*z* value of 1045.9 and an intensity higher than 634 and a peptide with an m/*z* value of 2517.6 and an intensity higher than 37 might be more likely to be in a caries-free state. The sensitivity to distinguish the changes in high-risk caries status was 84.5% using the decision tree model.

## Discussion

Early childhood caries is a crucial public health problem [18]. The latest epidemiological survey results in China demonstrated that the prevalence of deciduous dental caries in children aged 3–5 years was relatively high with an increasing trend, which was bound to have an immeasurable impact on physical and mental health, as well as socioeconomic development of these young populations. Saliva is one of the determinants of oral health, and its components, such as proteins and peptides, play an important role in maintaining oral health and tooth integrity. The diversity of salivary proteins among individuals is related to caries. Currently, screening people with a high-risk status of caries as early as possible is of vital significance for strengthening the comprehensive prevention of children's caries in implementing more accurate and effective prevention and intervention measures. The development of proteomic and peptidomic technologies has opened up horizons for dynamic monitoring of caries, and their advantages of high sensitivity and specificity provide a good rationale for exploring saliva biomarkers.

Caries risk means the probability that caries lesions will appear or progress if conditions remain the same within a stated period of time [19]. A 2-year follow-up of children found that having caries in the past was a great risk factor for new lesions (RR: 1.52, 95% CI 1.12–2.05) [20]. A prospective cohort study including 712 children in Beijing indicated that having previous caries was one of the important risk factors for the development of additional caries [21]. According to an early childhood caries risk category, a child under 6 years of age presenting with dentine cavities qualified as high risk [22]. According to reports in some studies, recurrent caries was identified in 37–54.2% of participants within 6 months after treatment for the first caries [23–25]. Therefore, the changes in caries status in Group C and Group CR in this study could be regarded as being at high risk of early childhood caries. A follow-up period of 6 months was necessary for monitoring caries changes in children. For the detection of predictive caries biomarkers before recurrence and to reduce the incidence of new lesions, the follow-up period was shortened to three months in this study.

Previous literature confirmed that salivary proteins were effective indicators for the evaluation of the development and progress of early childhood caries. Two peptides with *m*/*z* values of 1045.9 and 2517.6 screened in this study exhibited no significant difference in the cross-sectional comparison between different caries status groups and differential expression during dynamic changes of caries status, which indicated a close relationship between these two peptides and the dynamic changes of children's high-risk status of caries. Most of the previous studies of the same kind were cross-sectional comparative studies [26, 27], failing to reflect the causal relationship between biomarkers and diseases, which also showed a different trend in some cases among different study populations [28, 29]. All these studies indicated that it was necessary to introduce dynamic changes in caries status when considering biomarkers, as well as screen proteins and peptides with better specificity to assess changes in risk. As a longitudinally designed and regular follow-up study, the advantages were that it could combine the dynamic status of caries with the different expression of salivary proteins and peptides during the study period to determine biomarkers sensitively and effectively. Stimulated saliva was used as the collection method in the present study since it was a better option for preschool children who might have poorer compliance with the collection procedures of saliva samples, aiming to collect the sufficient volume of saliva sample more conveniently within a shorter time duration. Based on the findings of our pilot studies, the minimum interventive stimulation method (by brushing on children’s occlusal surfaces only) would not dramatically interfered with the analytical results of salivary peptidome and was already used in a series of previous studies conducted by our research group [17, 30, 31].

In this study, the statuses were divided into five types: health, untreated caries, caries after filling treatment, no caries after filling treatment, and missing teeth due to caries. During observation, there were no individuals with missing teeth due to caries, so we mainly investigated the mutual transformation relationship between the first four caries states in this study. According to the changes in caries status, subjects were divided into three groups that had no significant difference in sex or age at baseline. Previous studies have shown that some salivary proteins and peptides have differences between various caries statuses in children. Sun et al. [17] found 7 peptides in the saliva of patients with s-ECC before and after treatment, two of which were derived from histatin-1. Tian et al. [30] analysed saliva before treatment and at 10 days and 4 months after treatment and found that two peptides with m/z values of 3162.0 and 3290.4, respectively, were related to recurrence of s-ECC. In terms of the categories of changes in caries status, previous studies often divided subjects into groups DMFT (dmft) or the progress of caries [32]. Furthermore, some researchers refined the various classifications according to the International Caries Detection and Evaluation System (ICDAS). Guedes et al. [33] classified patients according to enamel caries and dentin caries and found that 8 salivary proteins in children’s saliva, including carbonic anhydrase 1, interleukin-36, and serum amyloid, were related to the occurrence of caries. To date, there have been very few reports that have considered both the history of caries treatment and recurrence experiences, as well as the variety of caries states, which was also a point of innovation of the present study.

For the two peptides found in this study (who were identified as segments of SMR-3B and mucin-7, respectively), no significant difference was detected between groups in diverse states, but they were differentially expressed among individuals’ transformations. SMR-3B is a protein with anti-inflammatory activity that belongs to the lipopolysaccharide-binding protein of *Aggregatibacter actinomycetemcomitans* and *Porphyromonas gingivali*s. It has the ability to maintain antimicrobial and immune regulation by interfering with bacterial adhesion and colonization [34, 35]. In in vitro tests, *A. actinomycetemcomitans* had an inhibitory effect on the growth of *Streptococcus mutans* [36]. During the process of caries status, *S. mutans* destroys the hard tissue of the tooth by synthesizing water-insoluble glucan to lower the local pH value [37, 38]. SMR-3B might increase the level of *Streptococcus mutans* in saliva by inhibiting the growth of *A. actinomycetemcomitans* and the associated risk of caries. Mucin is a leading part of innate immunity to maintain oral health and acts as a barrier against microorganisms and other adverse factors [39]. Mucin-7 combined with salivary proteins, such as proline-rich protein and statherin, was transported to the oral cavity to avoid proteolysis and improved the utilization of antibacterial proteins in the form of compounds. It could also be specifically united with proteins located on the surface of bacteria or directly induced aggregation in the mouth to remove cariogenic microorganisms [40]. Luo et al. [41] investigated 30 caries-susceptible and caries-free individuals and collected 5 min and 2 h of enamel-acquired biofilms for proteomics analysis. The results indicated that mucin-7 in the caries group had upregulated expression, but the trend was not affected by the time of acquired biofilm formation. A cross-sectional study of salivary protein in caries and caries-free adults found that the occurrence of caries was related to a decrease in mucin-7 levels. Mucin-7 could better distinguish between caries-carrying and caries-free states at a concentration of 2.5 ng/ml, which showed that the sensitivity and specificity were both 100% [42]. The changes in the concentration of salivary mucin mainly occurred before the eruption of deciduous teeth [43], which implied that when the oral cavity was in a healthy and stable state during the period of deciduous dentition, its concentration would not fluctuate significantly. Since mucins prevented the occurrence of dental caries by directly combining with microorganisms, mucin-7 in saliva could potentially affect the types of oral microbial colonization; therefore, it was also capable of reflecting the changes of oral microbes side-on. Nevertheless, one previous study [44] demonstrated that the concentration of mucin-7 in the saliva of preschool children was not much different across the various caries risks. From our findings, these two proteins (SMR-3B and mucin-7) showed no significant difference in cross-sectional comparison between the different groups, and they only occurred with the dynamically changed caries status of the individual. These results might be able to partly interpret the contradictions among previous studies.

Currently, the development of mass spectrometry technology has made it possible to measure protein expression levels simultaneously. However, the high-dimensional results made it difficult to visualize samples. The emergence of principal component analysis has simplified the data by dimensionality reduction; thus, it has been more intuitive to evaluate the differences and similarities between samples [45]. A principal component analysis model was established based on two peptides. The results showed that there was no obvious separated trend between groups at baseline, but the two groups, namely, those with caries experience and those without caries experience, showed a scattered trend after 3 months, which was increasingly significant over time. The decision tree model also reached similar conclusions. The two differential peptides with *m*/*z* values of 1045.9 and 2517.6, respectively, could better distinguish individuals with a high risk of caries who might be more likely to suffer from caries or relapse after treatment. Its sensitivity reached 84.6%, which had certain application value. These two differentially expressed peptides and their sources of proteins (SMR-3B and mucin-7) have great potential for monitoring dynamic changes in children's high risk of caries.

Certain limitations in this study should be considered before the extrapolation of these findings in the future. First, the sample size was slightly limited. For the participants who were kindergarten children, the reasons for moving home with their families and changing school or graduation during the follow-up period led to a reduction in the sample size at the 6-month follow-up, resulting in not strictly balanced number of participants in the three groups. In this case, some of the participants might not have samples of all the three time points involved, which would undoubtedly have some impact on the results though the participants were paired in age and gender in each group with no statistically significant difference with the intention of decreasing the impact of unbalanced sample size among different groups. Second, the MALDI-TOF MS technique was reported to have difficulty in detecting low-abundance peptides [46], but it remained an appropriate choice to be used in the present study for preliminary screening of peptidomic profiles. Also, protein concentrations were not precisely measured in this study, though some previous studies had brought forward the feasibility of using equal volumes of saliva with the same collection method for comparisons of peptide profiles between different samples using MALDI TOF MS [31, 47, 48]. It must be better if more precise quantification, standardisation and normalisation procedures were performed in subsequent analyses and if the overall protein concentration was taken into account during the conception and designing stage of the research, while it was expected that the detection and identification of peptides would certainly be more in-depth and accurate with the development of technology. Meanwhile, the employment of validation procedures of proteins (e.g. Western blotting and ELISA) would be certainly valuable to verify these candidate salivary biomarkers in future studies. Third, all the participants enrolled in the present study were from the same kindergarten and, therefore, not all characteristics of children in this age group might have been represented. As they were living in the same environment, consuming the same diet, and guided by the same oral hygiene instructions, the impact of some other confounding factors was significantly reduced, which was certainly advantageous in consideration of a relatively low level of interference. In future studies, an enlarged sample size, a prolonged follow-up period and the introduction of evolving techniques would be beneficial in verifying the findings of the present research and performing more in-depth investigations in this field.

## Conclusions

In summary, this study used a longitudinal regular follow-up design combined with mass spectrometry techniques to screen two specific proteins in children’s saliva, namely SMR-3B and mucin-7, which have the potentiality to serve as candidate biomarkers for dynamically monitoring the changes in children's high-risk status of caries.

## Data Availability

All data generated or analysed during this study are included in this published article.

## Abbreviations

MALDI-TOF MS: Matrix-assisted laser desorption-ionization time-of-flight mass spectrometry
LC-ESI-MS/MS: Liquid chromatography-electrospray ionization-tandem mass spectrometry
SMR-3B: Submaxillary gland androgen regulated protein 3B
PKUSSIRB: Peking University School (and Hospital) of Stomatology Institutional Review Board
SPSS: Statistical Product and Service Solutions
